# Conversion of extrinsic into intrinsic motivation and computer based testing (CBT)

**DOI:** 10.1186/s12909-018-1249-4

**Published:** 2018-06-19

**Authors:** Maral Hariri-Akbari, Behjat Shokrvash, Firooz Mahmoodi, Fatemeh Jahanjoo-Aminabad, Behzad Yousefi, Fariba Azabdaftari

**Affiliations:** 1 Department of Basic Sciences, Paramedical School, Tabriz University of Medical Sciences, Golgasht Ave, Tabriz, 5166615739 Iran; 2 Department of Health Education and Promotion, School of Health, Tabriz University of Medical Sciences, Golgasht Street, Tabriz, Iran; 3 Doctoral Candidate, Department of Education, School of Education and Psychology, Tabriz University, Tabriz, Iran; 4 Tehran University of Medical Sciences, Department of Biostatistics and Epidemiology, Tehran, Iran; 5 Paramedical School, Tabriz University of Medical Sciences, Tabriz, Iran

**Keywords:** Computer Based Testing, Motivation, Self-Determination Theory, Student, Iran

## Abstract

**Background:**

Because computers are used in many aspects of today’s life, it seems necessary to include them in teaching and assessment processes.

**Method:**

The aims of this cross-sectional study were to construct a multidimensional valid scale, to identify the factors that influenced the nature of student motivation on Computer Based Testing (CBT), to recognize how students self-regulated their activities around CBT, and to describe the efficiency of autonomous versus controlled situations on motivation. The study was carried out among 246 Iranian Paramedical Students of Tabriz Medical Sciences University, Tabriz, Iran; 2013–2014.

The researchers prepared a questionnaire, based on the Self-Determination Theory (SDT), containing 26 items with a five-point Likert scale. It was prepared according to a previous valid questionnaire and by sharing opinions with some students and five professors. The factor analysis was done to perform instructional and exploratory factor analysis.

**Results:**

The Kaiser-Meyer-Olkin(KMO) measure was performed and variables were correlated highly enough to provide a reasonable basis for factor analysis. The selected 4 factors determined a 60.28% of the variance; autonomy 26.37%, stimulation 14.11%, relatedness10.71%, and competency 9.10%.

**Conclusion:**

A questionnaire was prepared and validated, based on SDT variables. The results indicated that autonomous extrinsic motivation correlated positively with intrinsic motivation and CBT. There was a general positive attitude towards computer-based testing among students. As students became intrinsically motivated through the promotion of autonomous regulation, CBT was recommended as a proper test mode.

**Electronic supplementary material:**

The online version of this article (10.1186/s12909-018-1249-4) contains supplementary material, which is available to authorized users.

## Background

The past 45 years has been a period of significant reforms in the field of education, making assessment an integral part of the psychology of education [1, 2]. With increasing global use of computers for standardized testing purposes within high educational institutions [3–5], it is essential for today’s students to leave school with computer and information literacy skills that will enable them to adapt to a fast changing world [6] and avoid confusion when encountering these tests [7].

With flexibility in time and space, immediate scoring and feedback, and cost efficiency [8], CBT provides a strong intrinsic testing motivation [9]. A high level of student motivation has also been identified through computer-assisted instructions among senior medical students [10].

As a strong theory of motivation, the self-determination theory has proved to have both theoretical and practical potential for educational researchers and practitioners [11]. What SDT researchers are concerned about is the quality of motivation, intrinsic and extrinsic [11]. According to SDT, intrinsic motivation is a person’s most positive potential possession, an inherent capacity that makes people look for and learn new things and develop an integrative sense of self [12, 13]. In contrast to the idea that extrinsic rewards can undermine intrinsic motivation by diminishing autonomy [14], it is in encouraging circumstances that intrinsic motivation can find the opportunity to flourish [11]. An extrinsically motivated behavior can be converted into an intrinsic one through the promotion of autonomous regulations [15–17]. While many psychology researchers have become increasingly aware of the importance of cultural and contextual variables affecting students’ motivation, most of the research and attempts to create educational and technologic testing methods come from western countries, based on the needs and attitudes of Western students and overlook the needs and attitudes of Asian and Middle Eastern students [18]. Among the human subjects studied in a sample of papers chosen from the top psychology journals surveyed in 2008, 96% came from westernized industrial and wealthy societies, and 68% from the U.S.A., in particular [19].

Like many countries in the transitional world, Iranians also have to adopt themselves to the new technology which is based on computer application. Computers are used in every aspect of modern life, including education. Computers are usually used in classroom teaching and science seminars and conferences in Iran. However, it is not normally included in examination processes because of high cost and limited accessibility [20]. It is important to identify the factors that influence the motivation of Iranian students in applying CBT [4, 6, 21]. The present study aimed at constructing a valid scale to identify the kind of motivation and the factors that influence the attitude of Iranian students towards CBT, to explore how these students self-regulate the activities surrounding CBT through internalizing extrinsic into intrinsic motivation, and to assess the efficiency of autonomous versus controlled motivation among Iranian Paramedical Students.

## Methods

### Assessment development

A cross-sectional study carried out during the fall semester of 2013–2014 with the participation of 246 undergraduate paramedical students of the Tabriz Medical Sciences University taking General English in the fields of nursing, public health, food industries, laboratory sciences, anesthesia, and technology of radiology. All participants were provided with adequate information about the nature and reasons of the study, and were told in advance that the information they provided would be kept confidential. A consent form was signed by all the participants before the commencement of the study. The questionnaires were completed by the participants in class sessions, under the supervision of the researcher, using paper and pen. Adequate time (15–20 min) was allocated to all the participants to fill out the questionnaire. Their questions were answered and their doubts were clarified. Participants who were reluctant to submit the questionnaire after filling it out were excluded from the study. Then, the questionnaires were collected and subjected to a computer analysis. In analyzing the data, SPSS version 16 was used.

The questionnaire prepared by the researcher was based on SDT to estimate the quality of motivation (intrinsic and extrinsic) using a previous valid questionnaire [22]. It contained 26 items with a five-point Likert scale (ranging from strongly agree = 5, to strongly disagree = 1).

### Validity and reliability assessment

The reliability and validity of the questionnaire were determined. The qualitative content validity of the questionnaire was judged by five professors of social sciences, language education, statistics, and psychology, and the research deputy of the university. The opinions of some students were also sought in preparing the questionnaire.

### Factor analysis

A data reduction statistical technique was used that allowed the simplification of the correlation relationships between variables**.** Three steps; computation of the correlation metrics, the testing of the sampling adequacy, and the reduction of the number of variables; were taken to identify the important factors that influenced the attitude of students of the Tabriz Medical Sciences University towards CBT.

Kaiser-Meyer-Olkin (KMO) and Principal Component Analyses (PCA) were performed to indicate whether there was a reasonable basis for factor analysis and any interrelation between the questionnaire items. The factor analysis was done to perform instructional analysis, and an exploratory factor analysis was done to identify the dimensions of the scale. The reliability was calculated using Chronbach’s Alpha test.

## Results

### Sample characteristics

The number of 246undergraduate paramedical students, age ranging from 17 to 37 years, M (SD) 19.93 (2.02) participated in the study. All the participants had taken General English course in the fields of nursing, public health, food industries, laboratory sciences, anesthesia, and radiology at the Tabriz Medical Sciences University.

### Construct and validity of the questionnaire

No questions were excluded after the determination of the qualitative content validity of the scale, and all the 26 items remained. The PCA was performed to determine the interrelationship (s) between the questionnaire items. The value of the KMO [23] was measured and its significance value (*P* < 0.001) indicated that the variables correlated highly enough to provide a reasonable basis for factor analysis (Table 1).

**Table 1:** Kaiser-Meyer-Olkin and Bartlett’s Test results

KMO measure of sampling adequacy		.813
Bartlett’s Test of Sphericity	Approx. Chi-Square	2191.728
	df	325
	*P*-value	<.001

*Abbreviation: df* degree of freedom

Each factor had an Eigen-value> 1 for computer-based testing. The percentage of total variance was used as an index to determine how well the total factor solution accounted for the represented variables together. The index for the present solution accounted for 60.28%of the total variances for computer-based testing (Table 2). An exploratory factor analysis sorted the 26 questions into four factors, autonomy(7 items, variance% = 26.37), stimulation(10 items, variance% = 14.11),relatedness (3 items, variance% = 10.71), and competency(6 items, variance% = 9.10%)were extracted (Table 2). For example, among the items, item 2 was found to have the highest loading − 0.582, and item 6 the lowest loading with0.507 (Table 3).

**Table 2:** Initial Eigen values and extracted sums of squared loadings

Component	Initial Eigen values			Extraction sums of squared loadings		
	Total	% of Variance	Cumulative %	Total	% of Variance	Cumulative %
1	6.855	26.366	26.366	6.855	26.366	26.366
2	2.027	14.110	40.476	2.027	14.110	40.476
3	1.641	10.711	51.187	1.641	10.711	51.187
4	1.411	9.101	60.282	1.411	9.101	60.282
5	.987	5.277	60.282			
6	.985	3.735	65.559			
7	.984	3.368	69.294			
8	.973	2.48	72.662			
9	.928	2.369	75.142			
10	.866	2.361	77.511			
11	.787	2.326	79.872			
12	.771	2.224	82.198			
13	.710	2.173	84.422			
14	.655	1.517	86.595			
15	.584	1.447	88.112			
16	.529	1.336	89.559			
17	.488	1.275	90.895			
18	.479	1.141	92.17			
19	.443	1.105	93.311			
20	.383	1.102	94.416			
21	.346	.933	95.518			
22	.324	.845	96.451			
23	.304	.713	97.296			
24	.278	.697	98.009			
25	.258	.693	98.706			
26	.213	.601	100.00

**Table 3:** Items loaded into factors

Question number and item	Component				variance %
	1	2	3	4	
Autonomy					26.37
Q25 I like online testing because my exam sheet remains in my personal site and I can always review it	.582				
Q7 I like online testing because I feel I too have a personality of my own	.568				
Q26 I like online testing because I have a computer and privacy of my own while taking the test	.547				
Q11 I like online testing because it will provide me with the ability to start navigating on internet	.546				
Q8 I like online testing because It will help me to grow my individual identity	.529				
Q9 I like online testing because I can establish an organized file of my exams at university	.515				
Q6 I like online testing because It will help me to keep a personal site of my exams	.507				
Stimulation					14.11
Q1 I like online testing because l enjoy working with computer		.608			
Q21 I like online testing because the instructions will be given in a more transparent way		.567			
Q2 I like online testing because it is more pleasurable		.562			
Q14 I like online testing because it is easier to type on keyword rather than writing with pen or pencil		.543			
Q13 I like online testing because fewer trees will be cut to produce paper		.512			
Q 22 I like online testing because there will be minimum possibility of personal biasing in exam sheet correction		.492			
Q18 I like online testing because there will be little possibility of cheating on my paper		.492			
Q20 I like online testing because the exam sheets will be corrected in less time		.442			
Q19 I like online testing because my exam sheet will be corrected more accurately		.429			
Q12 I like online testing because less paper will be spared		.376			
Relatedness					10.71
Q5 I like online testing because It connects me to the global village			.525		
Q10 I like online testing because it is a window to the world of virtual reality			.492		
Q4 I like online testing because It makes me feel I am moving along with global trend			.472		
Competency					9.10
Q3 I like online testing because It makes me feel I am involved in an academic activity				.605	
Q17 I like online testing because I can learn from my mistakes				.604	
Q24 I like online testing because my computer based skills will be enhanced				.560	
Q23 I like online testing because my computer based knowledge will be enhanced				.486	
Q15 I like online testing because I later can search my exam sheet on the website easily				.478	
Q16 I like online testing because I can evaluate my exam sheet and find my mistakes				.475	

Abbreviation: *Q* question

A Pearson product-moment correlation coefficient was computed to assess the relationship between the 26-itemed questionnaire scale/subscales that each student had completed. The coefficient method indicated a positive interactive correlation between an increase in each factor with the increase in other factors and the total *p* < 0.001. (Table 4).

**Table 4:** Pearson product-moment correlation coefficient results

Factors		Stimulation	Competency	Autonomy	Relatedness
Stimulation	Pearson Correlation	1	.660^a^	.609^a^	.548^a^
	Sig. (2-tailed)		.001	.001	.001
	N	246	245	246	246
Competency	Pearson Correlation	.660^a^	1	.598^a^	.450^a^
	Sig. (2-tailed)	.000		.001	.001
	N	245	245	245	245
Autonomy	Pearson Correlation	.609^a^	.598^a^	1	.566^a^
	Sig. (2-tailed)	.001	.001		.001
	N	246	245	246	246
Relatedness	Pearson Correlation	.548^a^	.450^a^	.566^a^	1
	Sig. (2-tailed)	.001	.001	.001	
	N	246	245	246	246
Total	Pearson Correlation	.897^a^	.822^a^	.847^a^	.705^a^
	Sig. (2-tailed)	.001	.001	.001	.001
	N	245	245	245	245

*Abbreviation: Sig* significant ^a^correlation is significant at the 0.01 level (2-tailed)

As the number of factors were more than two, rotation was necessary to identify the differences between the initially extracted factors, and to provide a clear picture of items loaded on each factor. Table 5 shows Rotated Factor Matrix.

**Table 5:** Rotated Factor Matrix

Question number and item	Component				Variance %
	1	2	3	4	
Autonomy					26.37
Q 25 I like online testing because It remains in my personal site and I can always review it	.582				
Q 7 I like online testing because I feel I too have a personality of my own	.568				
Q 26 I like online testing because I have a computer and privacy of my while taking the test	.547				
Q11 I like online testing because it will provide with the ability to start navigating on internet	.546				
Q 8 I like online testing because It will help me to grow my individual identity	.529				
Q 9 I like online testing because I can establish an organized file of my exams at university	.515				
Q6 I like online testing because It will help me to keep a personal site of my exams	.507				
Stimulation					14.11
Q 1 I like online testing because l enjoy working with computer		.608			
Q 21 I like online testing because the instructions will be given in a more transparent way		.567			
Q 2 I like online testing because it is more pleasurable		.562			
Q14 I like online testing because it is easier to type on keyword rather than writing with pen or pencil		.543			
Q13 I like online testing because less trees will be cut to produce paper		.512			
Q 22 I like online testing because there will be minimum possibility of personal biasing in the correction		.492			
Q18 I like online testing because there is little possibility of cheating on my paper		.492			
Q 20 I like online testing because the exam sheets will be corrected in less time		.442			
Q 19 I like online testing because my exam sheet will be corrected more accurately		.429			
Q12 I like online testing because less paper will be spared		.376			
Relatedness					10.71
Q5. I like online testing because It connects me to the global village			.525		
Q10. I like online testing because it is a window to the world of virtual reality			.492		
Q4. I like online testing because It makes me feel I am moving along with global trend			.472		
Competency					9.10
Q3 I like online testing because It makes me feel I am involved in an academic activity				.605	
Q17 I like online testing because I can learn from my mistakes				.604	
Q 24 I like online testing because my computer based skills will be enhanced				.560	
Q 23 I like online testing because my computer based knowledge will be enhanced				.486	
Q15 I like online testing because I later can search my exam sheet on the website easily				.478	
Q16 I like online testing because I can evaluate my exam sheet and find my mistakes				.475	

Abbreviation: *Q* question

### Reliability of the questionnaire

The reliability of the questionnaire taken by the internal consistency of the 26 item questionnaire based on Chronbach’s Alpha was 0.884; and the factors of autonomy 0.749, Stimulation 0.719, Competency 0.709, and relatedness 0.557.

## Discussion

Based on the self-determination theory and its relevance to CBT, results of this study indicate that Iranian paramedical students were ready to convert their extrinsically motivated behavior into intrinsically motivated behavior by promoting autonomous regulations. This resulted from a general positive attitude among the students toward computer-based testing. In this study students converted their extrinsic motivation into intrinsic motivation based on the three basic human psychological needs of autonomy, relatedness, and competency, and one subcategory of intrinsic motivation, stimulation [24, 25]. The four above concepts formed the four factors of the study.

Autonomy with the highest degree of variance presented itself as the most paramount factor in growing self-esteem and identity (I like on line testing because I feel I too have a personality of my own: and online testing will help me to grow my individual identity). Therefore, factor-1 was entitled autonomy. The self-determination theory focuses on those aspects of social contexts that make the context more autonomy-supportive instead of being a controlling type [17].

Items in factor2 (stimulation), were concerned with the interest and excitement of the participants in working with computers, involving the items like (easier to type on a keyword than writing on paper, saving time and more accuracy and transparency in correcting exam sheets, less bias in correction, getting exam results soon), and environmental concerns such as (less trees will be cut and less paper will be used). Stimulation is one of the three sub-categories of intrinsic motivation. The other two are knowledge and accomplishment [24, 25]. The motivation associated with factors1and 2 in this study was extrinsic [11], which converted into intrinsic motivation through internalization regulation [17, 25].

Factor3 addressed relatedness, need to experience a sense of belonging, and attachment to others, people, and networks [17, 25]. As the technology of each age sets the mindset of the time [26], the Iranian student mindset of this age has a tendency toward using computers as a tool to become related to the current global trend, (I like CBT because I feel I am related to the global village, it connects me to the global village, a window to the world of virtual reality, and I move along with the global trend).

Factor4 dealt with competency and accomplishment, meaning, people are actively directed toward growth and meet challenges [17, 25]. Both factors 3 and 4 in this study reflected an introjected regulation because the students under study were proud of themselves and a feeling of worth led to the growth of their self-esteem [23, 27]. It is essential to mention that the items related to the motivation for knowledge (I like online testing because I can evaluate my exam sheet and find my mistakes; I can learn from my mistakes; and my computer-based knowledge will be enhanced), were categorized under the competency factor.

Based on the self-determination theory [23] and its relevance to CBT, extrinsic motivation through internalized (in factors1 and 2), and introjected (in factors3 and 4) regulations, correlated positively with intrinsic motivation and CBT among Iranian Premedical students in this study. There was a general positive attitude toward computer-based testing among the students.

Based on the self-determination theory, this study tried to evaluate the effects of autonomy-supportive contexts on the conversion of non-intrinsically motivated behaviors into intrinsically-motivated behaviors, and the way the feeling of having autonomy rather than being controlled, affected the attitude of the Tabriz Medical Sciences University students towards CBT. The regulation of behaviors towards CBT among Iranian students indicated its own specific characteristics. Compared with other students [28, 29], Iranian students approached CBT with a specific attitude due to their social and cultural moorings. As the degree of autonomy in initiating and accomplishing their activities is usually rather low and their choices to follow up their individual goals may be somewhat restricted, in the common context of the society they live in, Iranian students prefer to take their tests in a relatively autonomous rather than a controlled situation. This provides them with an opportunity to construct their motivation concerning CBT in terms of their self-esteem and identity.

Similar to the present research, a study at the University of Iowa, in the USA [30], and another in Malaysia [3] considered the use of educational technology as an improvement in educational quality, and a means of testing which of such technology can increase a participant’s intrinsic motivation and self-efficacy. Another study, also in Malaysia [31], reported a neutral attitude of the students toward web-based instruction. However, the study was in agreement with the present study with regard to students being stimulated by the task. The students in the study were strongly positive about the convenience of web-based instruction, because it provided them with the ability to control their pace of learning. According to a pedagogic study done in poor neighborhoods of the world for three decades, economic inequalities were seen as the major obstacle to access equality in education [32]. On the other hand, in an exploration of the degree of school administers’ interests by race, African American school administrators showed a stronger interest in developing and enhancing their abilities by using technology in performing tasks than did Caucasian school administrators [33]. Both studies were in agreement with this study, which took social context as a powerful factor in shaping student attitudes towards a testing mode.

The results of a study that found Iranian students’ perception on using computers mostly disadvantageous [34] came in contrast with the present study which found an overall positive student attitude towards CBT. It can partly relate to the nature of the studies. The previous study compared CBT with paper-based testing (PBT) in taking specific exams such as TOEFL and IELTS and evaluated the advantages and disadvantages of each testing mode over the other. However, the present research based on SDT, evaluated the general student perception on CBT and described how CBT induced the feeling of an autonomous versus controlled situation and caused students become intrinsically motivated while taking the test.

### Limitations

The present study has certain limitations:

- The computer related skills and literacy of the students were not evaluated before the distribution of the questionnaires.
- The sample size was relatively small, limited to the paramedical students.

## Conclusion

In this study, a 26-itemquestionnaire was prepared and validated, based on SDT variables. The results indicated Iranian students were stimulated by CBT, because they felt CBT would provide them with autonomy through which they would be able to demonstrate their competency, and working with computers would relate them to the global trends. Results also indicated autonomous extrinsic motivation correlated positively with intrinsic motivation and CBT. There was a general positive attitude toward computer-based testing among students. Because the Iranian students became intrinsically motivated through the promotion of autonomous regulation, the researchers suggest CBT as an alternative testing mode in taking exams in Iran.

Although the sample size was rather small, the study was the first of its kind to evaluate students’ attitude towards CBT in the Tabriz Medical Sciences University. The results may provide useful implications for teachers and the university. However, future research based on other theories of motivation with different variables can provide more helpful insights for higher educational centers and those who work on psychology of education.

## Additional files

Additional file 1: All raw data as supplementary information. (PDF 533 kb)

Additional file 2: Copy of questionnaire. (DOCX 19 kb)

## Abbreviation

BS: Batcher of Science
CBT: Computer Based Testing
KMO: Kaiser-Meyer Olkin
M(SD): Mean (Standard Deviation)
MPH: Master of Public Health
Ms.: Master of Science
N: Frequency
PCA: Principle Components Analysis
PhD: Doctor of Philosophy
SDT: Self-Determination Theory
Sig: Significance
