# Supplementary material for: Conversion of extrinsic into intrinsic motivation and computer based testing (CBT)
Source: BMC Med Educ. 2018 Jun 19;18:143. doi: 10.1186/s12909-018-1249-4 (PMC6006569; doi:10.1186/s12909-018-1249-4)
Supplement: Supplementary file 1 — All raw data as supplementary information. (PDF 533 kb) [file 12909_2018_1249_MOESM1_ESM.pdf]

gender	age	q1	q2	q3	q4	q5	q6	q7	q8	q9	
#NULL!	20.00	1.000	2.00	1.00	1.00	1.00	1.00	1.00	1.00	2.00	
2.00	20.00	4.000	4.00	3.00	1.00	1.00	1.00	1.00	1.00	1.00	
#NULL!	#NULL!	1.000	1.00	1.00	2.00	2.00	5.00	3.00	2.00	5.00	
2.00	19.00	3.000	5.00	4.00	3.00	2.00	1.00	1.00	3.00	3.00	
1.00	20.00	2.000	1.00	2.00	2.00	3.00	2.00	1.00	3.00	2.00	
2.00	20.00	3.000	2.00	2.00	1.00	1.00	1.00	3.00	2.00	2.00	
1.00	37.00	3.000	2.00	2.00	3.00	2.00	2.00	2.00	3.00	1.00	
2.00	21.00	3.000	3.00	2.00	2.00	2.00	3.00	3.00	3.00	2.00	
1.00	#NULL!	2.000	3.00	4.00	3.00	4.00	4.00	2.00	3.00	3.00	
2.00	18.00	3.000	3.00	2.00	2.00	3.00	3.00	3.00	4.00	4.00	
2.00	20.00	5.000	4.00	2.00	1.00	2.00	2.00	2.00	2.00	1.00	
1.00	18.00	1.000	2.00	3.00	1.00	2.00	2.00	1.00	1.00	2.00	
1.00	20.00	4.000	4.00	4.00	4.00	4.00	4.00	3.00	4.00	2.00	
1.00	21.00	3.000	3.00	3.00	3.00	3.00	4.00	3.00	3.00	1.00	
2.00	20.00	3.000	2.00	3.00	3.00	2.00	2.00	3.00	3.00	2.00	
2.00	23.00	2.000	1.00	3.00	1.00	1.00	2.00	4.00	3.00	3.00	
2.00	20.00	2.000	2.00	3.00	1.00	1.00	2.00	2.00	3.00	2.00	
2.00	19.00	3.000	2.00	2.00	2.00	2.00	1.00	3.00	3.00	2.00	
2.00	19.00	3.000	2.00	2.00	2.00	2.00	1.00	3.00	3.00	3.00	
2.00	19.00	2.000	3.00	2.00	2.00	2.00	2.00	2.00	3.00	2.00	
1.00	21.00	1.000	2.00	1.00	1.00	1.00	1.00	1.00	1.00	2.00	
2.00	18.00	4.000	3.00	2.00	3.00	4.00	4.00	3.00	2.00	1.00	
1.00	20.00	2.000	2.00	2.00	3.00	2.00	2.00	2.00	3.00	2.00	
1.00	19.00	1.000	2.00	1.00	1.00	1.00	1.00	1.00	1.00	2.00	
2.00	19.00	3.000	2.00	2.00	4.00	4.00	2.00	3.00	2.00	4.00	
1.00	27.00	1.000	1.00	2.00	2.00	2.00	3.00	2.00	2.00	2.00	
2.00	19.00	3.000	3.00	2.00	3.00	2.00	3.00	3.00	2.00	3.00	
2.00	20.00	1.000	1.00	2.00	2.00	2.00	4.00	4.00	1.00	2.00	
2.00	20.00	3.000	2.00	2.00	2.00	3.00	2.00	3.00	2.00	2.00	
2.00	19.00	3.000	3.00	2.00	1.00	1.00	5.00	3.00	1.00	1.00	
2.00	#NULL!	4.000	3.00	3.00	2.00	2.00	2.00	3.00	2.00	1.00	
2.00	19.00	1.000	2.00	1.00	1.00	1.00	2.00	1.00	1.00	2.00	
#NULL!	#NULL!	2.000	3.00	2.00	2.00	2.00	3.00	3.00	4.00	3.00	
2.00	19.00	3.000	2.00	4.00	4.00	4.00	3.00	4.00	4.00	3.00	
2.00	18.00	3.000	3.00	2.00	2.00	3.00	3.00	2.00	2.00	3.00	
2.00	19.00	2.000	2.00	2.00	1.00	3.00	3.00	3.00	2.00	4.00	

2.00	19.00	2.000	3.00	2.00	2.00	2.00	1.00	3.00	3.00	1.00
2.00	19.00	2.000	3.00	2.00	2.00	2.00	1.00	3.00	3.00	1.00
2.00	20.00	5.000	5.00	3.00	3.00	2.00	2.00	3.00	3.00	2.00
2.00	20.00	3.000	5.00	3.00	2.00	2.00	2.00	3.00	2.00	2.00
2.00	19.00	5.000	1.00	2.00	2.00	3.00	1.00	2.00	2.00	2.00
2.00	20.00	3.000	3.00	3.00	3.00	2.00	3.00	4.00	4.00	2.00
2.00	19.00	3.000	2.00	2.00	2.00	2.00	3.00	3.00	3.00	2.00
2.00	20.00	3.000	3.00	1.00	1.00	2.00	3.00	3.00	2.00	2.00
2.00	20.00	3.000	4.00	4.00	2.00	3.00	5.00	3.00	3.00	4.00
2.00	19.00	2.000	2.00	3.00	2.00	2.00	2.00	3.00	3.00	2.00
2.00	19.00	1.000	3.00	2.00	3.00	2.00	3.00	3.00	2.00	1.00
2.00	22.00	2.000	3.00	2.00	2.00	2.00	1.00	3.00	3.00	1.00
#NULL!	19.00	4.000	4.00	3.00	2.00	2.00	2.00	2.00	5.00	2.00
#NULL!	#NULL!	2.000	2.00	2.00	1.00	1.00	2.00	2.00	2.00	2.00
#NULL!	22.00	1.000	2.00	1.00	3.00	1.00	3.00	3.00	1.00	3.00
2.00	18.00	1.000	2.00	1.00	3.00	1.00	3.00	3.00	1.00	3.00
2.00	19.00	3.000	1.00	1.00	2.00	2.00	1.00	1.00	1.00	2.00
2.00	19.00	4.000	3.00	4.00	3.00	4.00	4.00	3.00	4.00	3.00
2.00	18.00	1.000	2.00	1.00	3.00	1.00	3.00	3.00	1.00	3.00
2.00	20.00	1.000	2.00	1.00	3.00	1.00	3.00	3.00	1.00	3.00
2.00	#NULL!	2.000	2.00	3.00	2.00	2.00	2.00	1.00	2.00	3.00
2.00	19.00	2.000	3.00	3.00	1.00	2.00	4.00	1.00	3.00	4.00
2.00	21.00	2.000	1.00	1.00	1.00	2.00	1.00	2.00	2.00	1.00
2.00	21.00	3.000	2.00	2.00	2.00	4.00	1.00	2.00	2.00	1.00
2.00	22.00	2.000	3.00	2.00	1.00	1.00	1.00	1.00	2.00	2.00
2.00	21.00	2.000	2.00	2.00	2.00	4.00	1.00	2.00	1.00	5.00
2.00	19.00	1.000	2.00	1.00	3.00	1.00	3.00	3.00	1.00	3.00
2.00	20.00	1.000	3.00	1.00	3.00	1.00	3.00	3.00	1.00	3.00
2.00	19.00	2.000	2.00	2.00	2.00	2.00	3.00	3.00	3.00	3.00
2.00	20.00	1.000	2.00	3.00	1.00	3.00	3.00	2.00	3.00	3.00
2.00	19.00	1.000	2.00	3.00	1.00	3.00	3.00	2.00	3.00	3.00
2.00	19.00	2.000	1.00	2.00	2.00	2.00	2.00	2.00	2.00	2.00
2.00	21.00	1.000	2.00	1.00	1.00	2.00	3.00	1.00	1.00	1.00
2.00	19.00	1.000	2.00	2.00	1.00	1.00	1.00	2.00	1.00	2.00
1.00	21.00	1.000	1.00	1.00	2.00	1.00	1.00	1.00	1.00	1.00
2.00	22.00	1.000	3.00	4.00	1.00	5.00	1.00	3.00	2.00	4.00
1.00	19.00	2.000	2.00	1.00	2.00	1.00	3.00	2.00	1.00	1.00

2.00	19.00	1.000	1.00	1.00	2.00	2.00	1.00	3.00	2.00	2.00
2.00	18.00	1.000	1.00	1.00	1.00	1.00	2.00	3.00	3.00	3.00
2.00	20.00	2.000	1.00	2.00	2.00	1.00	2.00	3.00	1.00	1.00
1.00	19.00	4.000	4.00	2.00	3.00	2.00	4.00	2.00	2.00	2.00
1.00	19.00	4.000	3.00	4.00	3.00	2.00	2.00	4.00	2.00	3.00
#NULL!	#NULL!	2.000	2.00	3.00	4.00	2.00	3.00	4.00	2.00	1.00
2.00	#NULL!	3.000	3.00	2.00	4.00	3.00	2.00	4.00	2.00	3.00
2.00	18.00	1.000	2.00	3.00	1.00	2.00	3.00	1.00	2.00	3.00
1.00	20.00	5.000	1.00	2.00	1.00	1.00	2.00	1.00	2.00	0.00
2.00	20.00	1.000	2.00	1.00	1.00	2.00	1.00	2.00	2.00	1.00
2.00	27.00	2.000	1.00	2.00	2.00	1.00	3.00	2.00	2.00	1.00
2.00	27.00	2.000	2.00	1.00	2.00	2.00	1.00	2.00	1.00	1.00
1.00	20.00	1.000	1.00	2.00	1.00	3.00	2.00	1.00	1.00	2.00
2.00	21.00	1.000	2.00	1.00	1.00	2.00	1.00	2.00	2.00	1.00
2.00	20.00	1.000	2.00	1.00	2.00	2.00	1.00	2.00	1.00	1.00
2.00	18.00	1.000	2.00	2.00	1.00	1.00	1.00	2.00	1.00	2.00
#NULL!	#NULL!	2.000	2.00	2.00	2.00	2.00	1.00	1.00	1.00	3.00
1.00	20.00	1.000	2.00	2.00	1.00	1.00	2.00	1.00	2.00	1.00
1.00	20.00	2.000	3.00	3.00	2.00	3.00	2.00	2.00	3.00	3.00
2.00	#NULL!	2.000	3.00	3.00	2.00	3.00	2.00	2.00	3.00	3.00
2.00	21.00	4.000	3.00	2.00	1.00	2.00	3.00	2.00	1.00	2.00
2.00	18.00	2.000	2.00	1.00	2.00	1.00	1.00	2.00	1.00	2.00
2.00	20.00	1.000	1.00	1.00	2.00	3.00	3.00	4.00	4.00	2.00
2.00	18.00	3.000	3.00	1.00	1.00	2.00	4.00	2.00	4.00	2.00
1.00	20.00	1.000	1.00	1.00	1.00	1.00	1.00	1.00	2.00	2.00
2.00	20.00	1.000	2.00	2.00	1.00	1.00	1.00	1.00	2.00	1.00
1.00	23.00	2.000	1.00	1.00	1.00	1.00	1.00	3.00	2.00	1.00
2.00	18.00	2.000	2.00	2.00	1.00	1.00	1.00	2.00	2.00	2.00
2.00	19.00	1.000	2.00	2.00	1.00	2.00	1.00	2.00	1.00	2.00
#NULL!	#NULL!	1.000	1.00	1.00	1.00	1.00	1.00	2.00	2.00	2.00
2.00	19.00	1.000	1.00	2.00	1.00	2.00	2.00	1.00	2.00	1.00
2.00	18.00	4.000	3.00	2.00	2.00	2.00	3.00	3.00	4.00	3.00
#NULL!	19.00	2.000	2.00	3.00	2.00	1.00	1.00	2.00	2.00	1.00
2.00	21.00	1.000	2.00	2.00	2.00	1.00	4.00	2.00	3.00	1.00
2.00	19.00	4.000	2.00	3.00	5.00	3.00	1.00	5.00	3.00	3.00
2.00	19.00	2.000	2.00	2.00	2.00	2.00	2.00	2.00	3.00	1.00
2.00	18.00	3.000	4.00	3.00	2.00	2.00	3.00	4.00	3.00	4.00

1.00	19.00	1.000	2.00	1.00	2.00	2.00	1.00	2.00	2.00	1.00
2.00	21.00	1.000	2.00	2.00	1.00	1.00	2.00	1.00	2.00	1.00
2.00	21.00	1.000	1.00	1.00	1.00	1.00	2.00	2.00	2.00	1.00
#NULL!	#NULL!	1.000	2.00	1.00	2.00	2.00	2.00	2.00	2.00	1.00
1.00	#NULL!	1.000	2.00	1.00	2.00	2.00	2.00	1.00	2.00	5.00
2.00	17.00	2.000	1.00	1.00	1.00	1.00	2.00	3.00	1.00	1.00
#NULL!	#NULL!	2.000	1.00	2.00	3.00	1.00	2.00	1.00	2.00	3.00
#NULL!	19.00	1.000	1.00	1.00	2.00	2.00	2.00	1.00	3.00	2.00
1.00	18.00	3.000	2.00	3.00	2.00	1.00	1.00	1.00	1.00	2.00
2.00	19.00	3.000	2.00	2.00	2.00	1.00	2.00	3.00	3.00	2.00
2.00	20.00	1.000	1.00	2.00	3.00	2.00	1.00	1.00	1.00	1.00
2.00	19.00	4.000	3.00	2.00	2.00	4.00	4.00	2.00	2.00	3.00
1.00	19.00	2.000	3.00	4.00	3.00	2.00	3.00	2.00	2.00	2.00
2.00	19.00	2.000	1.00	2.00	2.00	1.00	2.00	1.00	1.00	2.00
1.00	21.00	2.000	2.00	1.00	3.00	2.00	1.00	2.00	1.00	2.00
2.00	22.00	1.000	2.00	2.00	1.00	2.00	1.00	1.00	2.00	2.00
2.00	20.00	2.000	4.00	2.00	2.00	2.00	2.00	2.00	1.00	1.00
#NULL!	#NULL!	1.000	1.00	2.00	2.00	1.00	1.00	1.00	2.00	2.00
#NULL!	#NULL!	1.000	1.00	2.00	1.00	3.00	1.00	1.00	1.00	1.00
#NULL!	#NULL!	2.000	2.00	1.00	1.00	1.00	1.00	2.00	1.00	3.00
#NULL!	21.00	1.000	2.00	3.00	4.00	3.00	2.00	1.00	1.00	1.00
#NULL!	#NULL!	1.000	1.00	2.00	2.00	2.00	1.00	3.00	3.00	3.00
#NULL!	#NULL!	2.000	1.00	2.00	1.00	2.00	1.00	2.00	1.00	2.00
#NULL!	#NULL!	1.000	1.00	1.00	1.00	1.00	2.00	3.00	3.00	2.00
#NULL!	#NULL!	2.000	2.00	2.00	2.00	2.00	1.00	1.00	1.00	1.00
2.00	18.00	1.000	2.00	1.00	1.00	2.00	1.00	2.00	3.00	1.00
2.00	20.00	2.000	1.00	1.00	1.00	2.00	1.00	2.00	1.00	2.00
1.00	21.00	2.000	2.00	1.00	2.00	1.00	1.00	2.00	1.00	2.00
1.00	21.00	2.000	2.00	3.00	3.00	3.00	1.00	3.00	2.00	2.00
1.00	20.00	2.000	4.00	4.00	4.00	4.00	2.00	3.00	3.00	2.00
1.00	19.00	3.000	2.00	3.00	2.00	3.00	2.00	3.00	3.00	3.00
2.00	19.00	2.000	2.00	2.00	3.00	3.00	4.00	5.00	1.00	1.00
1.00	21.00	2.000	2.00	3.00	2.00	3.00	3.00	3.00	3.00	2.00
1.00	19.00	3.000	1.00	4.00	1.00	2.00	2.00	5.00	5.00	4.00
1.00	19.00	1.000	2.00	3.00	1.00	2.00	2.00	3.00	2.00	1.00
1.00	20.00	4.000	4.00	4.00	5.00	5.00	2.00	5.00	5.00	2.00
1.00	21.00	2.000	1.00	4.00	2.00	3.00	2.00	3.00	3.00	3.00

#NULL!	#NULL!	2.000	2.00	3.00	2.00	2.00	2.00	1.00	1.00	2.00
#NULL!	#NULL!	2.000	1.00	3.00	1.00	2.00	2.00	3.00	1.00	2.00
#NULL!	#NULL!	1.000	1.00	3.00	1.00	2.00	3.00	1.00	3.00	3.00
#NULL!	#NULL!	1.000	1.00	3.00	1.00	2.00	2.00	3.00	1.00	2.00
1.00	18.00	1.000	2.00	1.00	1.00	3.00	2.00	2.00	1.00	2.00
2.00	20.00	5.000	5.00	4.00	2.00	2.00	3.00	2.00	2.00	2.00
#NULL!	#NULL!	2.000	2.00	2.00	2.00	1.00	1.00	3.00	2.00	2.00
2.00	#NULL!	3.000	3.00	2.00	3.00	2.00	3.00	2.00	2.00	3.00
2.00	19.00	3.000	1.00	3.00	2.00	2.00	1.00	3.00	3.00	2.00
1.00	19.00	2.000	2.00	2.00	2.00	2.00	3.00	3.00	2.00	3.00
2.00	19.00	2.000	2.00	2.00	3.00	3.00	2.00	3.00	2.00	2.00
1.00	21.00	4.000	3.00	2.00	1.00	2.00	3.00	4.00	5.00	4.00
1.00	20.00	4.000	3.00	2.00	1.00	1.00	2.00	2.00	3.00	2.00
1.00	19.00	1.000	2.00	1.00	2.00	3.00	3.00	2.00	3.00	2.00
1.00	20.00	2.000	2.00	2.00	1.00	1.00	1.00	1.00	3.00	2.00
1.00	20.00	3.000	4.00	4.00	2.00	3.00	2.00	2.00	3.00	2.00
2.00	19.00	4.000	4.00	3.00	3.00	3.00	4.00	3.00	4.00	3.00
2.00	19.00	2.000	2.00	1.00	5.00	5.00	1.00	2.00	2.00	2.00
2.00	20.00	1.000	2.00	2.00	1.00	3.00	1.00	1.00	3.00	1.00
2.00	20.00	2.000	2.00	3.00	1.00	4.00	1.00	5.00	2.00	1.00
1.00	22.00	1.000	1.00	1.00	1.00	1.00	1.00	2.00	3.00	2.00
#NULL!	#NULL!	3.000	3.00	3.00	1.00	1.00	1.00	2.00	2.00	2.00
#NULL!	#NULL!	1.000	1.00	1.00	1.00	1.00	2.00	3.00	4.00	5.00
#NULL!	#NULL!	2.000	1.00	1.00	1.00	1.00	2.00	5.00	5.00	3.00
#NULL!	#NULL!	2.000	2.00	1.00	1.00	2.00	1.00	1.00	2.00	1.00
1.00	20.00	2.000	2.00	2.00	2.00	1.00	1.00	3.00	5.00	5.00
2.00	22.00	3.000	2.00	1.00	2.00	1.00	3.00	4.00	1.00	1.00
1.00	20.00	4.000	5.00	2.00	1.00	5.00	4.00	4.00	2.00	1.00
1.00	20.00	2.000	2.00	3.00	3.00	2.00	1.00	2.00	1.00	2.00
1.00	19.00	2.000	2.00	2.00	2.00	3.00	1.00	1.00	2.00	2.00
1.00	30.00	4.000	4.00	3.00	2.00	4.00	1.00	1.00	1.00	3.00
2.00	19.00	4.000	5.00	3.00	4.00	3.00	2.00	3.00	4.00	3.00
2.00	19.00	2.000	2.00	2.00	2.00	3.00	3.00	3.00	3.00	2.00
2.00	19.00	5.000	4.00	3.00	4.00	2.00	2.00	2.00	3.00	3.00
2.00	19.00	5.000	4.00	2.00	2.00	2.00	2.00	2.00	3.00	3.00
2.00	19.00	4.000	5.00	5.00	5.00	4.00	1.00	3.00	2.00	2.00
2.00	20.00	1.000	2.00	1.00	1.00	1.00	4.00	3.00	2.00	1.00

2.00	#NULL!	2.000	1.00	2.00	1.00	1.00	1.00	1.00	1.00	2.00
2.00	18.00	1.000	2.00	2.00	4.00	4.00	2.00	4.00	1.00	2.00
2.00	19.00	3.000	3.00	2.00	2.00	1.00	2.00	1.00	1.00	4.00
2.00	20.00	1.000	2.00	1.00	2.00	2.00	2.00	1.00	1.00	1.00
2.00	#NULL!	3.000	3.00	3.00	3.00	1.00	2.00	2.00	2.00	1.00
2.00	20.00	3.000	2.00	2.00	2.00	2.00	3.00	4.00	4.00	3.00
2.00	19.00	2.000	3.00	2.00	3.00	3.00	4.00	3.00	3.00	2.00
2.00	21.00	4.000	3.00	2.00	2.00	2.00	3.00	3.00	3.00	2.00
1.00	24.00	2.000	3.00	2.00	2.00	1.00	1.00	2.00	2.00	2.00
1.00	19.00	5.000	4.00	3.00	4.00	3.00	5.00	4.00	3.00	4.00
#NULL!	#NULL!	5.000	5.00	4.00	2.00	3.00	2.00	3.00	4.00	3.00
1.00	19.00	1.000	2.00	1.00	2.00	1.00	1.00	2.00	3.00	1.00
2.00	20.00	5.000	5.00	5.00	2.00	2.00	2.00	3.00	3.00	1.00
#NULL!	21.00	4.000	4.00	3.00	3.00	2.00	2.00	5.00	3.00	2.00
1.00	#NULL!	5.000	5.00	5.00	3.00	3.00	4.00	4.00	4.00	3.00
2.00	21.00	1.000	1.00	2.00	1.00	3.00	2.00	1.00	1.00	2.00
2.00	20.00	1.000	1.00	2.00	1.00	2.00	5.00	4.00	5.00	1.00
1.00	20.00	2.000	4.00	2.00	1.00	3.00	4.00	1.00	2.00	3.00
1.00	20.00	3.000	4.00	2.00	1.00	1.00	1.00	1.00	2.00	1.00
1.00	19.00	2.000	2.00	1.00	2.00	1.00	1.00	2.00	2.00	1.00
2.00	20.00	1.000	1.00	2.00	1.00	2.00	2.00	1.00	2.00	1.00
2.00	21.00	1.000	2.00	1.00	3.00	3.00	2.00	2.00	1.00	1.00
1.00	19.00	2.000	1.00	2.00	2.00	1.00	3.00	2.00	1.00	2.00
1.00	#NULL!	2.000	2.00	1.00	1.00	1.00	2.00	2.00	2.00	2.00
2.00	20.00	1.000	2.00	2.00	1.00	1.00	3.00	4.00	3.00	2.00
2.00	23.00	2.000	1.00	2.00	4.00	2.00	2.00	2.00	3.00	3.00
1.00	19.00	2.000	2.00	2.00	2.00	3.00	3.00	2.00	2.00	3.00
1.00	19.00	1.000	1.00	1.00	1.00	1.00	2.00	2.00	3.00	5.00
1.00	20.00	2.000	3.00	2.00	2.00	2.00	2.00	3.00	4.00	2.00
1.00	#NULL!	3.000	3.00	4.00	2.00	1.00	2.00	2.00	2.00	3.00
2.00	20.00	2.000	3.00	2.00	1.00	3.00	2.00	2.00	2.00	2.00
1.00	#NULL!	3.000	3.00	2.00	1.00	1.00	2.00	2.00	1.00	2.00
2.00	19.00	2.000	2.00	1.00	2.00	1.00	1.00	2.00	1.00	2.00
#NULL!	#NULL!	2.000	3.00	4.00	5.00	3.00	3.00	2.00	2.00	2.00
2.00	19.00	1.000	1.00	1.00	2.00	1.00	2.00	1.00	2.00	1.00
2.00	20.00	2.000	1.00	2.00	2.00	1.00	2.00	1.00	2.00	2.00
2.00	20.00	2.000	2.00	1.00	2.00	1.00	1.00	2.00	3.00	1.00

2.00	21.00	1.000	4.00	2.00	3.00	1.00	3.00	1.00	2.00	1.00
2.00	20.00	1.000	2.00	2.00	2.00	1.00	1.00	5.00	5.00	5.00
1.00	22.00	1.000	1.00	2.00	5.00	5.00	5.00	2.00	2.00	1.00
1.00	18.00	1.000	1.00	1.00	2.00	1.00	1.00	1.00	2.00	1.00
1.00	23.00	2.000	3.00	4.00	1.00	1.00	1.00	2.00	3.00	3.00
#NULL!	19.00	1.000	2.00	3.00	4.00	3.00	2.00	1.00	2.00	3.00
1.00	18.00	1.000	1.00	1.00	1.00	1.00	2.00	2.00	2.00	3.00
2.00	20.00	1.000	1.00	2.00	2.00	1.00	1.00	1.00	3.00	3.00
#NULL!	#NULL!	5.000	5.00	5.00	5.00	5.00	5.00	5.00	5.00	5.00
1.00	20.00	1.000	1.00	2.00	1.00	2.00	2.00	1.00	2.00	1.00
1.00	19.00	1.000	1.00	2.00	1.00	2.00	1.00	1.00	2.00	1.00
2.00	21.00	1.000	1.00	3.00	3.00	2.00	2.00	2.00	3.00	1.00
1.00	20.00	1.000	1.00	1.00	2.00	2.00	1.00	2.00	2.00	3.00
1.00	19.00	1.000	1.00	1.00	1.00	1.00	2.00	3.00	3.00	3.00
#NULL!	#NULL!	1.000	1.00	1.00	1.00	1.00	1.00	1.00	1.00	1.00
1.00	18.00	4.000	3.00	2.00	1.00	2.00	2.00	1.00	2.00	1.00
#NULL!	#NULL!	2.000	2.00	2.00	2.00	2.00	2.00	2.00	2.00	2.00
2.00	20.00	2.000	2.00	1.00	2.00	1.00	1.00	2.00	1.00	2.00
1.00	19.00	2.000	2.00	2.00	2.00	3.00	2.00	2.00	1.00	4.00
2.00	22.00	3.000	2.00	4.00	3.00	4.00	2.00	4.00	1.00	3.00
2.00	19.00	1.000	2.00	3.00	3.00	2.00	2.00	4.00	1.00	5.00
2.00	18.00	5.000	5.00	5.00	5.00	5.00	5.00	5.00	5.00	5.00
1.00	20.00	2.000	3.00	2.00	2.00	3.00	3.00	3.00	2.00	3.00
1.00	21.00	2.000	3.00	2.00	2.00	2.00	2.00	1.00	2.00	2.00
1.00	17.00	2.000	1.00	1.00	2.00	1.00	2.00	1.00	2.00	1.00

q10	q11	q12	q13	q14	q15	q16	q17	q18	q19	q20
1.00	1.00	1.00	2.00	2.00	2.00	1.00	1.00	1.00	1.00	5.00
2.00	2.00	2.00	2.00	4.00	2.00	2.00	2.00	2.00	2.00	3.00
1.00	2.00	5.00	3.00	5.00	5.00	4.00	3.00	2.00	2.00	2.00
1.00	3.00	1.00	1.00	5.00	1.00	1.00	4.00	3.00	3.00	1.00
1.00	3.00	2.00	1.00	2.00	1.00	2.00	1.00	2.00	1.00	2.00
3.00	3.00	1.00	1.00	4.00	3.00	3.00	4.00	3.00	2.00	1.00
2.00	2.00	1.00	1.00	4.00	3.00	2.00	2.00	2.00	4.00	1.00
3.00	3.00	3.00	2.00	4.00	2.00	3.00	3.00	1.00	1.00	1.00
2.00	4.00	0.00	2.00	2.00	2.00	2.00	3.00	4.00	2.00	2.00
2.00	2.00	1.00	1.00	3.00	4.00	2.00	2.00	3.00	3.00	3.00
2.00	2.00	4.00	2.00	5.00	2.00	2.00	2.00	1.00	3.00	4.00
1.00	1.00	1.00	1.00	2.00	1.00	1.00	1.00	2.00	1.00	1.00
2.00	2.00	1.00	1.00	5.00	2.00	1.00	3.00	4.00	1.00	5.00
2.00	1.00	2.00	1.00	1.00	1.00	2.00	2.00	5.00	3.00	1.00
2.00	3.00	2.00	2.00	3.00	3.00	2.00	2.00	3.00	2.00	2.00
2.00	4.00	1.00	1.00	2.00	2.00	4.00	4.00	4.00	2.00	1.00
3.00	3.00	1.00	1.00	1.00	2.00	2.00	2.00	1.00	1.00	1.00
2.00	2.00	1.00	1.00	3.00	2.00	2.00	2.00	2.00	2.00	2.00
3.00	2.00	2.00	2.00	3.00	2.00	2.00	1.00	2.00	3.00	2.00
3.00	2.00	2.00	2.00	3.00	2.00	2.00	1.00	3.00	2.00	2.00
1.00	1.00	1.00	2.00	2.00	2.00	1.00	1.00	1.00	1.00	2.00
3.00	3.00	1.00	1.00	4.00	4.00	2.00	2.00	4.00	5.00	1.00
2.00	3.00	1.00	1.00	2.00	1.00	2.00	2.00	3.00	2.00	1.00
1.00	1.00	1.00	1.00	2.00	2.00	1.00	1.00	1.00	1.00	2.00
2.00	3.00	3.00	3.00	3.00	2.00	3.00	2.00	4.00	2.00	3.00
1.00	1.00	1.00	3.00	3.00	1.00	2.00	2.00	2.00	3.00	2.00
3.00	3.00	2.00	2.00	3.00	3.00	3.00	2.00	3.00	3.00	2.00
1.00	1.00	4.00	4.00	4.00	1.00	1.00	1.00	1.00	2.00	1.00
3.00	3.00	1.00	1.00	4.00	1.00	1.00	1.00	1.00	1.00	1.00
3.00	5.00	1.00	1.00	5.00	3.00	1.00	1.00	5.00	5.00	1.00
1.00	1.00	1.00	1.00	5.00	3.00	2.00	2.00	2.00	2.00	1.00
3.00	1.00	1.00	1.00	3.00	1.00	1.00	1.00	1.00	1.00	1.00
2.00	3.00	1.00	1.00	2.00	2.00	3.00	4.00	2.00	1.00	2.00
3.00	3.00	2.00	2.00	4.00	2.00	2.00	3.00	2.00	2.00	2.00
2.00	2.00	2.00	1.00	3.00	2.00	3.00	2.00	2.00	3.00	2.00
4.00	1.00	1.00	1.00	4.00	4.00	2.00	2.00	3.00	3.00	1.00

2.00	2.00	1.00	1.00	4.00	2.00	2.00	2.00	3.00	3.00	1.00
2.00	2.00	1.00	1.00	4.00	2.00	2.00	2.00	3.00	3.00	1.00
2.00	2.00	1.00	1.00	3.00	2.00	2.00	1.00	4.00	1.00	3.00
1.00	1.00	1.00	1.00	4.00	2.00	1.00	2.00	3.00	2.00	1.00
4.00	4.00	4.00	4.00	1.00	1.00	1.00	1.00	5.00	5.00	2.00
4.00	4.00	1.00	1.00	3.00	2.00	2.00	2.00	3.00	3.00	2.00
2.00	2.00	2.00	1.00	4.00	3.00	2.00	2.00	2.00	3.00	3.00
4.00	4.00	1.00	1.00	4.00	1.00	1.00	2.00	2.00	3.00	4.00
3.00	2.00	1.00	1.00	5.00	2.00	4.00	4.00	3.00	4.00	2.00
2.00	2.00	1.00	1.00	5.00	2.00	2.00	2.00	5.00	1.00	1.00
3.00	1.00	1.00	1.00	5.00	2.00	1.00	1.00	3.00	2.00	1.00
2.00	2.00	1.00	1.00	4.00	2.00	2.00	4.00	3.00	4.00	2.00
2.00	2.00	1.00	1.00	3.00	2.00	2.00	2.00	5.00	1.00	3.00
3.00	2.00	1.00	1.00	1.00	2.00	3.00	4.00	3.00	2.00	1.00
1.00	3.00	1.00	1.00	4.00	1.00	2.00	1.00	2.00	2.00	1.00
1.00	3.00	1.00	1.00	4.00	1.00	2.00	1.00	3.00	3.00	1.00
1.00	1.00	1.00	2.00	1.00	2.00	2.00	2.00	1.00	1.00	1.00
4.00	3.00	4.00	4.00	4.00	3.00	4.00	4.00	4.00	3.00	3.00
1.00	3.00	1.00	1.00	4.00	1.00	2.00	1.00	3.00	3.00	1.00
1.00	3.00	1.00	1.00	4.00	1.00	2.00	1.00	3.00	3.00	1.00
2.00	1.00	1.00	1.00	3.00	2.00	2.00	2.00	2.00	2.00	2.00
1.00	1.00	1.00	1.00	1.00	5.00	5.00	5.00	4.00	5.00	5.00
2.00	2.00	1.00	1.00	2.00	1.00	1.00	1.00	2.00	2.00	1.00
4.00	3.00	1.00	1.00	5.00	3.00	2.00	2.00	1.00	2.00	1.00
1.00	2.00	1.00	1.00	2.00	1.00	1.00	1.00	2.00	1.00	1.00
3.00	3.00	1.00	1.00	5.00	3.00	2.00	#NULL!	1.00	2.00	1.00
1.00	3.00	1.00	1.00	4.00	1.00	2.00	1.00	3.00	3.00	1.00
1.00	3.00	1.00	1.00	4.00	1.00	2.00	1.00	3.00	3.00	1.00
3.00	3.00	3.00	2.00	2.00	2.00	2.00	2.00	2.00	2.00	2.00
1.00	3.00	1.00	1.00	1.00	2.00	2.00	2.00	1.00	1.00	1.00
1.00	1.00	1.00	1.00	1.00	2.00	2.00	2.00	1.00	1.00	1.00
2.00	2.00	2.00	2.00	2.00	2.00	2.00	2.00	2.00	2.00	2.00
1.00	2.00	2.00	2.00	1.00	1.00	2.00	2.00	2.00	1.00	1.00
1.00	1.00	1.00	1.00	1.00	1.00	1.00	2.00	1.00	1.00	1.00
1.00	1.00	2.00	1.00	1.00	1.00	1.00	1.00	1.00	5.00	1.00
1.00	3.00	4.00	3.00	1.00	3.00	3.00	5.00	2.00	1.00	5.00
2.00	2.00	1.00	2.00	1.00	2.00	2.00	1.00	2.00	1.00	1.00

1.00	1.00	1.00	2.00	2.00	2.00	2.00	2.00	1.00	1.00	1.00
2.00	2.00	1.00	1.00	1.00	2.00	2.00	2.00	2.00	1.00	1.00
2.00	1.00	2.00	1.00	1.00	2.00	1.00	2.00	2.00	1.00	2.00
4.00	2.00	1.00	1.00	2.00	1.00	1.00	2.00	1.00	1.00	1.00
3.00	1.00	3.00	2.00	4.00	3.00	2.00	4.00	3.00	2.00	4.00
2.00	3.00	4.00	3.00	2.00	3.00	4.00	2.00	1.00	2.00	2.00
4.00	2.00	4.00	3.00	2.00	1.00	3.00	4.00	5.00	3.00	2.00
1.00	2.00	3.00	5.00	1.00	1.00	1.00	3.00	4.00	2.00	5.00
3.00	1.00	3.00	1.00	1.00	2.00	5.00	3.00	1.00	2.00	1.00
2.00	1.00	2.00	2.00	1.00	2.00	1.00	2.00	1.00	1.00	2.00
2.00	1.00	2.00	1.00	1.00	1.00	2.00	1.00	2.00	1.00	2.00
2.00	1.00	2.00	1.00	1.00	2.00	1.00	2.00	1.00	2.00	1.00
1.00	2.00	1.00	1.00	1.00	1.00	2.00	1.00	2.00	3.00	2.00
2.00	1.00	1.00	2.00	1.00	2.00	1.00	2.00	1.00	2.00	1.00
2.00	1.00	2.00	2.00	1.00	2.00	2.00	1.00	2.00	1.00	2.00
1.00	2.00	1.00	1.00	2.00	2.00	1.00	1.00	1.00	2.00	2.00
3.00	3.00	2.00	2.00	1.00	1.00	1.00	2.00	2.00	2.00	3.00
1.00	2.00	1.00	1.00	1.00	2.00	1.00	2.00	1.00	2.00	2.00
2.00	2.00	2.00	1.00	2.00	3.00	2.00	2.00	3.00	2.00	2.00
2.00	2.00	3.00	3.00	3.00	2.00	3.00	2.00	2.00	2.00	2.00
2.00	3.00	2.00	2.00	2.00	2.00	2.00	3.00	1.00	2.00	2.00
1.00	2.00	2.00	1.00	1.00	2.00	2.00	1.00	2.00	1.00	2.00
1.00	1.00	1.00	1.00	4.00	4.00	5.00	2.00	1.00	4.00	1.00
3.00	2.00	1.00	1.00	5.00	4.00	3.00	3.00	3.00	4.00	4.00
1.00	1.00	2.00	1.00	3.00	5.00	5.00	1.00	1.00	1.00	1.00
2.00	2.00	1.00	1.00	2.00	2.00	2.00	1.00	1.00	1.00	1.00
1.00	2.00	1.00	1.00	2.00	2.00	1.00	2.00	1.00	2.00	2.00
3.00	1.00	2.00	1.00	1.00	1.00	1.00	1.00	2.00	3.00	5.00
1.00	2.00	1.00	5.00	5.00	5.00	5.00	5.00	5.00	5.00	5.00
1.00	2.00	1.00	1.00	1.00	1.00	1.00	1.00	1.00	1.00	2.00
1.00	2.00	2.00	1.00	1.00	2.00	1.00	2.00	2.00	1.00	2.00
3.00	4.00	4.00	2.00	4.00	4.00	2.00	3.00	2.00	3.00	2.00
3.00	3.00	1.00	1.00	3.00	1.00	3.00	1.00	1.00	1.00	1.00
2.00	1.00	1.00	2.00	1.00	1.00	1.00	2.00	2.00	2.00	1.00
3.00	4.00	1.00	4.00	2.00	2.00	2.00	2.00	2.00	2.00	2.00
3.00	3.00	1.00	1.00	3.00	2.00	2.00	1.00	3.00	3.00	1.00
4.00	3.00	1.00	1.00	3.00	2.00	2.00	3.00	3.00	2.00	2.00

2.00	1.00	2.00	1.00	1.00	2.00	2.00	1.00	2.00	2.00	1.00
3.00	2.00	1.00	2.00	1.00	1.00	2.00	1.00	2.00	2.00	1.00
3.00	1.00	2.00	1.00	1.00	1.00	1.00	2.00	2.00	3.00	1.00
3.00	3.00	1.00	1.00	1.00	1.00	2.00	1.00	2.00	3.00	2.00
5.00	2.00	2.00	2.00	1.00	2.00	3.00	4.00	3.00	2.00	2.00
1.00	1.00	2.00	3.00	1.00	1.00	2.00	2.00	1.00	1.00	2.00
2.00	2.00	1.00	3.00	2.00	1.00	3.00	2.00	1.00	2.00	1.00
1.00	2.00	3.00	1.00	1.00	1.00	1.00	3.00	2.00	3.00	2.00
1.00	2.00	3.00	2.00	2.00	1.00	2.00	1.00	1.00	2.00	1.00
3.00	2.00	1.00	1.00	4.00	2.00	1.00	1.00	5.00	2.00	1.00
2.00	2.00	2.00	2.00	2.00	1.00	2.00	1.00	1.00	2.00	2.00
3.00	2.00	1.00	1.00	5.00	3.00	2.00	2.00	2.00	2.00	3.00
1.00	2.00	2.00	2.00	2.00	3.00	2.00	2.00	2.00	3.00	2.00
1.00	1.00	2.00	1.00	2.00	2.00	1.00	1.00	2.00	1.00	2.00
1.00	2.00	1.00	2.00	1.00	1.00	2.00	1.00	2.00	1.00	2.00
1.00	1.00	2.00	1.00	1.00	2.00	1.00	2.00	1.00	2.00	1.00
3.00	1.00	1.00	1.00	1.00	1.00	1.00	2.00	2.00	2.00	2.00
2.00	1.00	1.00	1.00	2.00	2.00	2.00	2.00	3.00	1.00	1.00
2.00	1.00	1.00	2.00	2.00	1.00	3.00	2.00	2.00	1.00	1.00
3.00	5.00	5.00	5.00	1.00	2.00	2.00	3.00	2.00	2.00	3.00
1.00	2.00	1.00	2.00	1.00	1.00	1.00	1.00	2.00	2.00	2.00
3.00	2.00	1.00	1.00	1.00	1.00	2.00	2.00	2.00	1.00	1.00
1.00	1.00	2.00	1.00	1.00	1.00	1.00	1.00	1.00	1.00	1.00
2.00	1.00	1.00	1.00	1.00	2.00	2.00	1.00	2.00	1.00	2.00
1.00	3.00	2.00	2.00	2.00	2.00	1.00	1.00	1.00	1.00	2.00
2.00	1.00	2.00	1.00	2.00	3.00	2.00	1.00	1.00	2.00	3.00
2.00	2.00	3.00	5.00	1.00	1.00	1.00	1.00	1.00	1.00	3.00
2.00	1.00	2.00	1.00	1.00	2.00	1.00	1.00	2.00	1.00	1.00
3.00	2.00	1.00	1.00	2.00	2.00	1.00	2.00	3.00	2.00	2.00
3.00	3.00	3.00	1.00	5.00	2.00	4.00	3.00	5.00	2.00	2.00
3.00	3.00	2.00	3.00	2.00	2.00	3.00	3.00	5.00	2.00	2.00
3.00	4.00	5.00	5.00	5.00	2.00	2.00	3.00	1.00	1.00	1.00
3.00	4.00	1.00	1.00	4.00	2.00	2.00	2.00	3.00	2.00	2.00
2.00	4.00	1.00	1.00	4.00	2.00	1.00	2.00	3.00	1.00	1.00
1.00	2.00	1.00	1.00	2.00	2.00	1.00	1.00	1.00	2.00	2.00
2.00	3.00	1.00	1.00	5.00	2.00	2.00	4.00	2.00	2.00	2.00
2.00	4.00	1.00	2.00	4.00	5.00	2.00	1.00	4.00	1.00	1.00

2.00	2.00	1.00	1.00	4.00	2.00	2.00	2.00	2.00	2.00	2.00
1.00	2.00	4.00	4.00	1.00	2.00	2.00	3.00	4.00	4.00	1.00
2.00	2.00	4.00	4.00	1.00	2.00	2.00	3.00	4.00	4.00	1.00
1.00	2.00	4.00	4.00	1.00	2.00	2.00	3.00	4.00	4.00	1.00
1.00	1.00	2.00	1.00	1.00	2.00	1.00	2.00	1.00	2.00	1.00
2.00	2.00	1.00	1.00	2.00	3.00	2.00	2.00	1.00	2.00	1.00
2.00	2.00	2.00	1.00	5.00	1.00	2.00	2.00	5.00	2.00	1.00
3.00	3.00	3.00	3.00	4.00	2.00	2.00	2.00	3.00	3.00	2.00
1.00	2.00	2.00	3.00	3.00	2.00	3.00	2.00	2.00	2.00	2.00
2.00	3.00	2.00	2.00	3.00	2.00	3.00	2.00	2.00	2.00	2.00
3.00	2.00	2.00	2.00	4.00	3.00	2.00	2.00	2.00	2.00	2.00
3.00	3.00	4.00	1.00	4.00	4.00	3.00	3.00	1.00	4.00	4.00
1.00	2.00	1.00	1.00	2.00	1.00	2.00	1.00	3.00	1.00	2.00
4.00	2.00	1.00	5.00	2.00	3.00	2.00	2.00	3.00	2.00	4.00
2.00	1.00	3.00	4.00	3.00	2.00	2.00	3.00	2.00	2.00	2.00
2.00	2.00	2.00	2.00	4.00	2.00	3.00	2.00	2.00	2.00	2.00
2.00	2.00	1.00	1.00	5.00	5.00	3.00	3.00	3.00	3.00	3.00
1.00	1.00	5.00	5.00	1.00	2.00	5.00	5.00	5.00	1.00	1.00
1.00	3.00	1.00	1.00	3.00	2.00	2.00	2.00	2.00	2.00	2.00
5.00	2.00	1.00	1.00	5.00	1.00	1.00	1.00	3.00	3.00	3.00
2.00	2.00	1.00	1.00	2.00	1.00	2.00	3.00	2.00	1.00	1.00
4.00	4.00	4.00	1.00	3.00	1.00	3.00	1.00	3.00	1.00	2.00
4.00	3.00	2.00	1.00	1.00	2.00	3.00	2.00	1.00	3.00	2.00
2.00	1.00	1.00	1.00	1.00	2.00	1.00	2.00	1.00	2.00	3.00
2.00	3.00	3.00	5.00	1.00	2.00	3.00	4.00	5.00	4.00	3.00
4.00	3.00	2.00	1.00	1.00	2.00	1.00	2.00	1.00	2.00	1.00
2.00	1.00	3.00	1.00	4.00	5.00	2.00	1.00	1.00	1.00	1.00
2.00	2.00	1.00	1.00	4.00	2.00	3.00	4.00	1.00	2.00	2.00
2.00	2.00	1.00	1.00	4.00	2.00	2.00	2.00	1.00	2.00	2.00
3.00	4.00	1.00	1.00	5.00	4.00	2.00	2.00	1.00	1.00	1.00
1.00	2.00	1.00	1.00	4.00	1.00	1.00	1.00	1.00	1.00	1.00
3.00	5.00	2.00	2.00	4.00	2.00	2.00	2.00	2.00	2.00	2.00
3.00	2.00	2.00	1.00	3.00	2.00	2.00	1.00	1.00	1.00	2.00
2.00	4.00	4.00	2.00	4.00	4.00	2.00	2.00	2.00	2.00	2.00
2.00	4.00	1.00	1.00	5.00	2.00	1.00	1.00	1.00	1.00	1.00
3.00	3.00	2.00	2.00	4.00	3.00	2.00	2.00	3.00	3.00	2.00
1.00	1.00	2.00	2.00	2.00	1.00	1.00	1.00	2.00	2.00	2.00

2.00	1.00	1.00	1.00	2.00	2.00	1.00	1.00	1.00	1.00	3.00
4.00	2.00	1.00	1.00	1.00	3.00	2.00	2.00	1.00	1.00	4.00
4.00	1.00	1.00	1.00	2.00	2.00	2.00	1.00	1.00	1.00	1.00
1.00	1.00	1.00	5.00	3.00	2.00	2.00	1.00	1.00	2.00	1.00
1.00	1.00	1.00	2.00	1.00	2.00	3.00	3.00	3.00	2.00	2.00
3.00	4.00	1.00	1.00	4.00	2.00	3.00	3.00	2.00	2.00	2.00
2.00	1.00	2.00	2.00	4.00	4.00	3.00	2.00	2.00	2.00	2.00
3.00	3.00	1.00	1.00	4.00	2.00	3.00	3.00	2.00	2.00	2.00
2.00	1.00	2.00	2.00	4.00	2.00	2.00	2.00	3.00	2.00	2.00
3.00	3.00	3.00	3.00	4.00	3.00	4.00	4.00	5.00	3.00	4.00
4.00	5.00	2.00	2.00	5.00	2.00	1.00	2.00	3.00	2.00	4.00
1.00	2.00	1.00	1.00	1.00	1.00	2.00	1.00	1.00	2.00	1.00
2.00	2.00	1.00	1.00	5.00	2.00	2.00	2.00	2.00	2.00	2.00
2.00	2.00	5.00	2.00	4.00	2.00	2.00	4.00	2.00	2.00	2.00
3.00	4.00	4.00	2.00	5.00	2.00	2.00	2.00	2.00	2.00	2.00
1.00	2.00	2.00	3.00	2.00	1.00	2.00	1.00	2.00	2.00	1.00
1.00	1.00	2.00	1.00	1.00	2.00	1.00	2.00	3.00	5.00	1.00
2.00	2.00	2.00	1.00	3.00	3.00	2.00	1.00	1.00	1.00	1.00
2.00	2.00	1.00	1.00	2.00	1.00	1.00	1.00	1.00	1.00	1.00
2.00	1.00	2.00	1.00	1.00	2.00	2.00	1.00	2.00	2.00	1.00
1.00	2.00	1.00	2.00	1.00	2.00	1.00	1.00	2.00	1.00	1.00
1.00	2.00	2.00	2.00	1.00	2.00	2.00	2.00	2.00	2.00	2.00
2.00	1.00	2.00	1.00	1.00	2.00	2.00	1.00	1.00	2.00	1.00
2.00	1.00	1.00	1.00	3.00	3.00	2.00	2.00	2.00	2.00	2.00
2.00	2.00	1.00	1.00	4.00	2.00	1.00	1.00	1.00	2.00	1.00
3.00	3.00	1.00	1.00	4.00	2.00	2.00	1.00	3.00	2.00	1.00
2.00	3.00	2.00	1.00	4.00	1.00	1.00	1.00	2.00	1.00	3.00
5.00	1.00	2.00	1.00	5.00	1.00	1.00	5.00	1.00	1.00	3.00
3.00	2.00	1.00	1.00	4.00	2.00	2.00	3.00	3.00	4.00	2.00
3.00	3.00	2.00	1.00	2.00	3.00	2.00	3.00	3.00	3.00	3.00
2.00	2.00	3.00	4.00	4.00	2.00	3.00	4.00	3.00	2.00	1.00
3.00	3.00	2.00	1.00	2.00	2.00	1.00	3.00	1.00	2.00	3.00
2.00	1.00	1.00	2.00	1.00	2.00	1.00	2.00	1.00	2.00	1.00
2.00	2.00	2.00	2.00	5.00	4.00	2.00	3.00	3.00	4.00	2.00
1.00	1.00	1.00	1.00	1.00	2.00	3.00	3.00	3.00	1.00	1.00
1.00	1.00	2.00	2.00	1.00	1.00	2.00	1.00	2.00	1.00	1.00
2.00	1.00	2.00	2.00	1.00	1.00	2.00	1.00	2.00	1.00	2.00

1.00	1.00	3.00	2.00	1.00	1.00	1.00	3.00	2.00	2.00	1.00
5.00	5.00	5.00	1.00	1.00	2.00	3.00	2.00	1.00	2.00	3.00
1.00	1.00	1.00	1.00	2.00	2.00	2.00	2.00	2.00	3.00	3.00
2.00	1.00	2.00	1.00	1.00	1.00	2.00	1.00	2.00	1.00	2.00
1.00	1.00	1.00	1.00	1.00	2.00	3.00	3.00	2.00	1.00	1.00
4.00	3.00	2.00	1.00	1.00	1.00	1.00	1.00	1.00	1.00	1.00
1.00	1.00	2.00	2.00	2.00	2.00	2.00	1.00	3.00	1.00	1.00
5.00	5.00	4.00	1.00	2.00	2.00	2.00	2.00	2.00	2.00	2.00
5.00	5.00	5.00	5.00	1.00	1.00	1.00	1.00	1.00	1.00	1.00
1.00	2.00	1.00	2.00	1.00	2.00	1.00	1.00	2.00	1.00	2.00
2.00	2.00	1.00	2.00	1.00	2.00	2.00	1.00	1.00	2.00	1.00
1.00	1.00	1.00	1.00	1.00	2.00	2.00	2.00	2.00	2.00	3.00
3.00	4.00	4.00	1.00	1.00	2.00	2.00	2.00	5.00	4.00	3.00
1.00	1.00	1.00	1.00	1.00	2.00	2.00	2.00	2.00	3.00	1.00
1.00	1.00	1.00	1.00	1.00	2.00	2.00	1.00	1.00	2.00	2.00
2.00	2.00	1.00	1.00	1.00	2.00	2.00	2.00	3.00	3.00	3.00
2.00	2.00	2.00	2.00	2.00	1.00	1.00	1.00	1.00	1.00	1.00
2.00	1.00	1.00	2.00	1.00	2.00	1.00	1.00	2.00	2.00	1.00
3.00	1.00	1.00	1.00	5.00	2.00	3.00	2.00	2.00	2.00	2.00
2.00	4.00	3.00	1.00	3.00	2.00	3.00	4.00	2.00	3.00	2.00
2.00	3.00	4.00	2.00	1.00	2.00	2.00	3.00	5.00	4.00	3.00
5.00	5.00	5.00	5.00	4.00	4.00	5.00	5.00	5.00	5.00	5.00
3.00	3.00	1.00	1.00	4.00	3.00	3.00	2.00	2.00	2.00	2.00
2.00	2.00	2.00	2.00	2.00	1.00	1.00	1.00	2.00	2.00	3.00
1.00	1.00	1.00	1.00	1.00	1.00	2.00	2.00	1.00	1.00	1.00

q21	q22	q23	q24	q25	q26
3.00	1.00	1.00	3.00	1.00	1.00
2.00	3.00	3.00	3.00	2.00	2.00
2.00	1.00	2.00	1.00	3.00	3.00
2.00	3.00	3.00	3.00	3.00	1.00
1.00	2.00	2.00	2.00	1.00	2.00
1.00	2.00	1.00	1.00	1.00	2.00
2.00	1.00	1.00	1.00	2.00	1.00
1.00	1.00	3.00	3.00	3.00	3.00
2.00	3.00	4.00	3.00	3.00	2.00
3.00	3.00	2.00	2.00	4.00	4.00
2.00	2.00	2.00	2.00	2.00	2.00
1.00	1.00	1.00	1.00	1.00	1.00
1.00	3.00	1.00	5.00	2.00	2.00
2.00	3.00	1.00	1.00	1.00	2.00
2.00	2.00	2.00	3.00	2.00	2.00
2.00	3.00	4.00	4.00	3.00	2.00
2.00	3.00	3.00	3.00	2.00	2.00
2.00	3.00	2.00	1.00	2.00	1.00
2.00	3.00	3.00	2.00	2.00	3.00
3.00	2.00	1.00	1.00	2.00	3.00
1.00	2.00	2.00	1.00	1.00	1.00
2.00	3.00	4.00	5.00	1.00	1.00
1.00	2.00	2.00	2.00	2.00	2.00
1.00	2.00	2.00	1.00	1.00	1.00
3.00	4.00	3.00	5.00	4.00	5.00
1.00	3.00	2.00	1.00	2.00	3.00
2.00	2.00	2.00	2.00	2.00	3.00
2.00	3.00	3.00	1.00	2.00	2.00
3.00	1.00	2.00	2.00	1.00	1.00
1.00	1.00	1.00	1.00	3.00	1.00
3.00	2.00	2.00	2.00	3.00	3.00
2.00	2.00	3.00	3.00	2.00	2.00
3.00	1.00	3.00	2.00	3.00	2.00
2.00	3.00	3.00	3.00	2.00	3.00
1.00	1.00	2.00	2.00	3.00	1.00
3.00	2.00	2.00	2.00	4.00	4.00

2.00	2.00	2.00	2.00	2.00	2.00
2.00	2.00	2.00	2.00	2.00	2.00
4.00	2.00	2.00	2.00	2.00	2.00
3.00	2.00	2.00	2.00	2.00	1.00
2.00	2.00	4.00	4.00	4.00	4.00
3.00	1.00	2.00	2.00	3.00	3.00
3.00	3.00	2.00	2.00	2.00	2.00
4.00	2.00	3.00	2.00	3.00	3.00
3.00	3.00	2.00	2.00	5.00	4.00
3.00	2.00	2.00	2.00	2.00	4.00
3.00	1.00	1.00	1.00	3.00	3.00
2.00	3.00	1.00	1.00	4.00	4.00
2.00	2.00	1.00	1.00	3.00	3.00
2.00	3.00	4.00	5.00	4.00	2.00
1.00	3.00	4.00	1.00	1.00	3.00
1.00	3.00	4.00	1.00	1.00	3.00
2.00	1.00	1.00	1.00	2.00	1.00
3.00	4.00	3.00	4.00	3.00	4.00
1.00	3.00	4.00	1.00	1.00	3.00
1.00	3.00	4.00	1.00	1.00	3.00
2.00	2.00	3.00	3.00	1.00	3.00
3.00	4.00	5.00	5.00	4.00	3.00
1.00	2.00	1.00	1.00	2.00	2.00
3.00	2.00	2.00	2.00	4.00	2.00
2.00	2.00	2.00	1.00	2.00	2.00
3.00	2.00	2.00	2.00	4.00	5.00
1.00	3.00	4.00	1.00	1.00	3.00
1.00	3.00	4.00	1.00	1.00	3.00
2.00	2.00	2.00	2.00	2.00	2.00
3.00	1.00	2.00	3.00	2.00	3.00
3.00	1.00	2.00	3.00	2.00	3.00
2.00	2.00	2.00	2.00	2.00	2.00
1.00	1.00	2.00	1.00	1.00	1.00
2.00	1.00	2.00	1.00	1.00	2.00
1.00	1.00	1.00	1.00	1.00	1.00
4.00	2.00	4.00	2.00	5.00	1.00
2.00	1.00	2.00	1.00	2.00	2.00

3.00	2.00	1.00	1.00	1.00	2.00
3.00	3.00	1.00	2.00	1.00	1.00
1.00	2.00	1.00	2.00	2.00	2.00
2.00	4.00	4.00	3.00	2.00	4.00
2.00	4.00	2.00	4.00	2.00	2.00
2.00	1.00	2.00	3.00	4.00	4.00
3.00	4.00	2.00	1.00	3.00	3.00
5.00	3.00	2.00	3.00	5.00	5.00
2.00	5.00	3.00	2.00	2.00	2.00
1.00	2.00	2.00	1.00	2.00	2.00
1.00	2.00	1.00	2.00	1.00	2.00
2.00	1.00	2.00	1.00	2.00	2.00
2.00	1.00	2.00	1.00	2.00	2.00
1.00	2.00	1.00	2.00	1.00	1.00
1.00	2.00	1.00	2.00	1.00	1.00
1.00	2.00	2.00	1.00	1.00	1.00
3.00	3.00	1.00	1.00	1.00	1.00
1.00	2.00	1.00	3.00	2.00	2.00
2.00	1.00	2.00	3.00	2.00	2.00
3.00	2.00	3.00	2.00	2.00	2.00
3.00	2.00	2.00	2.00	3.00	2.00
2.00	1.00	2.00	1.00	2.00	2.00
5.00	2.00	4.00	1.00	3.00	3.00
4.00	3.00	4.00	4.00	4.00	4.00
1.00	2.00	1.00	2.00	1.00	1.00
1.00	1.00	2.00	2.00	1.00	1.00
2.00	1.00	2.00	1.00	1.00	2.00
5.00	5.00	3.00	2.00	1.00	1.00
3.00	2.00	1.00	1.00	2.00	2.00
2.00	2.00	1.00	1.00	1.00	3.00
1.00	1.00	2.00	1.00	2.00	2.00
4.00	1.00	1.00	1.00	2.00	2.00
2.00	1.00	2.00	2.00	1.00	1.00
1.00	1.00	1.00	2.00	2.00	1.00
3.00	1.00	2.00	4.00	3.00	3.00
3.00	3.00	2.00	2.00	1.00	2.00
3.00	2.00	2.00	3.00	2.00	2.00

1.00	2.00	1.00	2.00	1.00	1.00
1.00	2.00	1.00	2.00	1.00	1.00
1.00	1.00	2.00	1.00	2.00	1.00
1.00	1.00	2.00	1.00	2.00	1.00
3.00	2.00	1.00	2.00	1.00	1.00
1.00	2.00	1.00	2.00	2.00	2.00
2.00	1.00	3.00	3.00	2.00	1.00
1.00	2.00	2.00	1.00	2.00	3.00
2.00	1.00	2.00	2.00	1.00	1.00
2.00	1.00	2.00	2.00	2.00	2.00
1.00	1.00	2.00	3.00	1.00	1.00
3.00	2.00	2.00	2.00	3.00	3.00
2.00	1.00	2.00	2.00	1.00	1.00
2.00	1.00	2.00	1.00	2.00	2.00
2.00	1.00	2.00	1.00	2.00	2.00
2.00	1.00	1.00	1.00	2.00	2.00
3.00	4.00	3.00	2.00	1.00	1.00
1.00	1.00	1.00	1.00	2.00	2.00
1.00	1.00	2.00	1.00	2.00	1.00
1.00	1.00	1.00	1.00	1.00	1.00
3.00	3.00	3.00	2.00	1.00	1.00
1.00	2.00	3.00	3.00	2.00	1.00
1.00	1.00	1.00	1.00	1.00	1.00
2.00	1.00	3.00	3.00	5.00	5.00
3.00	2.00	3.00	2.00	1.00	1.00
2.00	1.00	2.00	1.00	1.00	2.00
2.00	3.00	1.00	1.00	2.00	2.00
2.00	1.00	1.00	1.00	2.00	2.00
3.00	2.00	2.00	2.00	2.00	2.00
3.00	1.00	2.00	2.00	2.00	3.00
3.00	2.00	2.00	3.00	2.00	3.00
3.00	1.00	4.00	4.00	3.00	3.00
2.00	1.00	2.00	2.00	2.00	2.00
4.00	1.00	1.00	1.00	1.00	3.00
1.00	1.00	1.00	1.00	1.00	1.00
4.00	1.00	1.00	1.00	1.00	3.00
3.00	1.00	3.00	1.00	1.00	5.00

3.00	1.00	2.00	2.00	2.00	2.00
4.00	2.00	3.00	4.00	2.00	1.00
4.00	3.00	3.00	4.00	2.00	1.00
4.00	2.00	3.00	4.00	2.00	1.00
2.00	1.00	2.00	1.00	2.00	1.00
1.00	1.00	2.00	2.00	2.00	2.00
4.00	2.00	1.00	1.00	1.00	4.00
2.00	3.00	2.00	2.00	2.00	3.00
1.00	3.00	2.00	2.00	1.00	3.00
2.00	2.00	4.00	2.00	3.00	2.00
3.00	1.00	1.00	1.00	2.00	1.00
4.00	4.00	4.00	3.00	4.00	1.00
3.00	3.00	3.00	3.00	2.00	2.00
5.00	1.00	2.00	4.00	1.00	4.00
2.00	1.00	2.00	1.00	1.00	2.00
3.00	2.00	2.00	2.00	2.00	2.00
4.00	1.00	4.00	3.00	4.00	4.00
2.00	3.00	2.00	2.00	2.00	2.00
3.00	1.00	3.00	3.00	2.00	2.00
1.00	5.00	2.00	2.00	1.00	3.00
1.00	2.00	1.00	2.00	1.00	1.00
2.00	2.00	2.00	2.00	2.00	2.00
1.00	3.00	2.00	1.00	2.00	2.00
2.00	1.00	2.00	2.00	1.00	1.00
2.00	1.00	1.00	1.00	1.00	1.00
3.00	2.00	1.00	2.00	1.00	1.00
1.00	1.00	2.00	3.00	2.00	1.00
3.00	3.00	2.00	1.00	5.00	4.00
2.00	3.00	3.00	3.00	2.00	1.00
3.00	1.00	2.00	1.00	2.00	2.00
1.00	1.00	2.00	3.00	1.00	1.00
3.00	2.00	3.00	3.00	2.00	3.00
2.00	1.00	1.00	1.00	2.00	2.00
1.00	1.00	1.00	1.00	3.00	4.00
2.00	1.00	3.00	3.00	2.00	5.00
2.00	2.00	2.00	3.00	3.00	2.00
1.00	1.00	2.00	2.00	1.00	1.00

2.00	1.00	2.00	2.00	1.00	1.00
1.00	3.00	1.00	2.00	1.00	1.00
4.00	1.00	1.00	1.00	2.00	2.00
2.00	1.00	2.00	1.00	1.00	1.00
1.00	2.00	2.00	2.00	1.00	1.00
2.00	2.00	3.00	3.00	4.00	3.00
2.00	1.00	2.00	2.00	2.00	3.00
2.00	2.00	3.00	3.00	2.00	2.00
2.00	2.00	1.00	1.00	1.00	2.00
3.00	4.00	5.00	4.00	2.00	2.00
3.00	2.00	3.00	3.00	2.00	3.00
1.00	1.00	1.00	1.00	1.00	1.00
2.00	3.00	2.00	2.00	2.00	2.00
3.00	3.00	2.00	2.00	3.00	4.00
3.00	2.00	2.00	2.00	2.00	3.00
1.00	2.00	2.00	1.00	2.00	2.00
2.00	1.00	2.00	2.00	1.00	1.00
1.00	1.00	2.00	2.00	2.00	2.00
1.00	1.00	2.00	2.00	1.00	1.00
2.00	1.00	2.00	1.00	2.00	2.00
2.00	2.00	1.00	2.00	2.00	2.00
2.00	1.00	1.00	2.00	1.00	1.00
2.00	1.00	2.00	1.00	2.00	2.00
1.00	1.00	1.00	1.00	1.00	1.00
2.00	2.00	2.00	2.00	1.00	1.00
2.00	2.00	1.00	2.00	2.00	1.00
2.00	1.00	1.00	1.00	4.00	1.00
5.00	1.00	2.00	3.00	5.00	5.00
3.00	2.00	2.00	3.00	5.00	5.00
3.00	3.00	2.00	2.00	2.00	3.00
1.00	1.00	2.00	2.00	2.00	2.00
3.00	2.00	1.00	1.00	1.00	2.00
1.00	2.00	1.00	1.00	2.00	2.00
3.00	2.00	2.00	2.00	2.00	1.00
1.00	1.00	1.00	1.00	2.00	1.00
2.00	1.00	2.00	1.00	2.00	2.00
1.00	2.00	1.00	1.00	2.00	2.00

1.00	2.00	2.00	1.00	2.00	2.00
2.00	1.00	2.00	3.00	1.00	1.00
2.00	1.00	1.00	2.00	1.00	1.00
1.00	3.00	1.00	1.00	1.00	1.00
1.00	1.00	1.00	1.00	1.00	1.00
2.00	3.00	4.00	5.00	4.00	3.00
1.00	1.00	1.00	1.00	2.00	3.00
1.00	3.00	3.00	1.00	2.00	5.00
1.00	1.00	1.00	1.00	1.00	1.00
2.00	1.00	2.00	1.00	2.00	2.00
1.00	2.00	1.00	2.00	1.00	1.00
3.00	3.00	2.00	1.00	1.00	1.00
2.00	1.00	1.00	1.00	1.00	1.00
1.00	1.00	1.00	2.00	1.00	1.00
1.00	1.00	2.00	2.00	2.00	2.00
2.00	2.00	2.00	2.00	2.00	2.00
1.00	1.00	1.00	1.00	1.00	1.00
1.00	2.00	1.00	2.00	2.00	2.00
2.00	2.00	2.00	2.00	2.00	2.00
5.00	1.00	1.00	4.00	3.00	3.00
2.00	3.00	2.00	2.00	1.00	2.00
5.00	5.00	5.00	5.00	5.00	5.00
3.00	3.00	3.00	2.00	3.00	3.00
3.00	2.00	2.00	2.00	2.00	2.00
2.00	1.00	1.00	1.00	1.00	1.00
