# Supplementary material for: Conversion of extrinsic into intrinsic motivation and computer based testing (CBT)
Source: BMC Med Educ. 2018 Jun 19;18:143. doi: 10.1186/s12909-018-1249-4 (PMC6006569; doi:10.1186/s12909-018-1249-4)
Supplement: Supplementary file 2 — Copy of questionnaire. (DOCX 19 kb) [file 12909_2018_1249_MOESM2_ESM.docx]

**Student Attitude Questionnaire on**

**Computer Based Testing**

**Date…/…/…**

**Dear student,**

This questionnaire is prepared to assess your attitude on computer based testing. The information you provide will remain anonymous. Taking part in the survey is optional. If you wish to participate in the study, please complete the questionnaire.

**Student demographic data**

**Gender: Male Female**

**Age: ….**

**Field of study:**

Public health Food industries Anesthesia Nursing

Laboratory sciences Technology of radiology Midwifery

**Mother Language**

Farsi Turkish Kurdish Arabic other….

**Student Attitude Questionnaire**

**Please respond to the statements below by ticking appropriate box**

| **Questions** |  |  |  |  |  |
| --- | --- | --- | --- | --- | --- |
| 1. I like online testing because l enjoy working with computer | Strongly agree | Agree | No idea | Disagree | Strongly disagree |
| 2. I like online testing because it is more pleasurable | Strongly agree | Agree | No idea | Disagree | Strongly disagree |
| 3. I like online testing because It makes me feel I am involved in an academic activity | Strongly agree | Agree | No idea | Disagree | Strongly disagree |
| 4. I like online testing because It makes me feel I am moving along with global trend | Strongly agree | Agree | No idea | Disagree | Strongly disagree |
| 5. I like online testing because It connects me to the global village | Strongly agree | Agree | No idea | Disagree | Strongly disagree |
| 6. I like online testing because It will help me to keep a personal site of my exams | Strongly agree | Agree | No idea | Disagree | Strongly disagree |
| 7. I like online testing because I feel I too have a personality of my own | Strongly agree | Agree | No idea | Disagree | Strongly disagree |
| 8. I like online testing because It will help me to grow my individual identity | Strongly agree | Agree | No idea | Disagree | Strongly disagree |
| 9.I like online testing because I can establish an organized file of my exams at university | Strongly agree | Agree | No idea | Disagree | Strongly disagree |
| 10. I like online testing because it is a window to the world of virtual reality | Strongly agree | Agree | No idea | Disagree | Strongly disagree |
| 11. I like online testing because it will provide me with the ability to start navigating on internet | Strongly agree | Agree | No idea | Disagree | Strongly disagree |
| 12. I like online testing because less paper will be spared | Strongly agree | Agree | No idea | Disagree | Strongly disagree |
| 13. I like online testing because fewer trees will be cut to produce paper | Strongly agree | Agree | No idea | Disagree | Strongly disagree |
| 14. I like online testing because it is easier to type on keyword rather than writing with pen or pencil | Strongly agree | Agree | No idea | Disagree | Strongly disagree |

**Student Attitude Questionnaire**

**Please respond to the statements below by ticking appropriate box**

| **Questions** |  |  |  |  |  |
| --- | --- | --- | --- | --- | --- |
| 15. I like online testing because I later can search my exam sheet on the website easily | Strongly agree | Agree | No idea | Disagree | Strongly disagree |
| 16. I like online testing because I can evaluate my exam sheet and find my mistakes | Strongly agree | Agree | No idea | Disagree | Strongly disagree |
| 17. I like online testing because I can learn from my mistakes | Strongly agree | Agree | No idea | Disagree | Strongly disagree |
| 18. I like online testing because there will be little possibility of cheating on my paper | Strongly agree | Agree | No idea | Disagree | Strongly disagree |
| 19. I like online testing because my exam sheet will be corrected more accurately | Strongly agree | Agree | No idea | Disagree | Strongly disagree |
| 20. I like online testing because the exam sheets will be corrected in less time | Strongly agree | Agree | No idea | Disagree | Strongly disagree |
| 21. I like online testing because the instructions will be given in a more transparent way | Strongly agree | Agree | No idea | Disagree | Strongly disagree |
| 22. I like online testing because there will be minimum possibility of personal biasing in exam sheet correction | Strongly agree | Agree | No idea | Disagree | Strongly disagree |
| 23. I like online testing because my computer based knowledge will be enhanced | Strongly agree | Agree | No idea | Disagree | Strongly disagree |
| 24. I like online testing because my computer based skills will be enhanced | Strongly agree | Agree | No idea | Disagree | Strongly disagree |
| 25. I like online testing because my exam sheet remains in my personal site and I can always review it | Strongly agree | Agree | No idea | Disagree | Strongly disagree |
| 26. I like online testing because I have a computer and privacy of my own while taking the test | Strongly agree | Agree | No idea | Disagree | Strongly disagree |
